# Post-Colonization Interval Estimates Using Multi-Species Calliphoridae Larval Masses and Spatially Distinct Temperature Data Sets: A Case Study

**DOI:** 10.3390/insects8020040

**Published:** 2017-04-04

**Authors:** Courtney R. Weatherbee, Jennifer L. Pechal, Trevor Stamper, M. Eric Benbow

**Affiliations:** 1 Department of Entomology, Michigan State University, 243 Natural Science Building, East Lansing, MI 48824, USA; weath108@msu.edu (C.R.W.); pechalje@msu.edu (J.L.P.); 2 Department of Entomology, Purdue University, 901 West State Street, West Lafayette, IN 47907, USA; tstampe@purdue.edu; 3 Department of Osteopathic Medical Specialties, Michigan State University, 243 Natural Science Building, East Lansing, MI 48824, USA; 4 Ecology, Evolutionary Biology and Behavior, Michigan State University, 243 Natural Science Building, East Lansing, MI 48824, USA

**Keywords:** forensic entomology, decomposition, variability, necrobiome, post-mortem interval, *Phormia regina*, *Cochliomyia macellaria*

## Abstract

Common forensic entomology practice has been to collect the largest Diptera larvae from a scene and use published developmental data, with temperature data from the nearest weather station, to estimate larval development time and post-colonization intervals (PCIs). To evaluate the accuracy of PCI estimates among Calliphoridae species and spatially distinct temperature sources, larval communities and ambient air temperature were collected at replicate swine carcasses (N = 6) throughout decomposition. Expected accumulated degree hours (ADH) associated with *Cochliomyia macellaria* and *Phormia regina* third instars (presence and length) were calculated using published developmental data sets. Actual ADH ranges were calculated using temperatures recorded from multiple sources at varying distances (0.90 m–7.61 km) from the study carcasses: individual temperature loggers at each carcass, a local weather station, and a regional weather station. Third instars greatly varied in length and abundance. The expected ADH range for each species successfully encompassed the average actual ADH for each temperature source, but overall under-represented the range. For both calliphorid species, weather station data were associated with more accurate PCI estimates than temperature loggers associated with each carcass. These results provide an important step towards improving entomological evidence collection and analysis techniques, and developing forensic error rates.

## 1. Introduction

Determining time of death is a significant element of solving crimes, yet it is a difficult piece of information to estimate using existing, and often fragmented, evidence collected during a death scene investigation. Patterns of postmortem insect succession and development are common tools used to help narrow the estimated time since death, a time range commonly referred to as the postmortem interval (PMI) [1,2]. Indeed, insects provide valuable clues towards narrowing the estimate of the time since death, however, the term PMI can be misleading as it implies the entire time elapsed since death, and not just the insect activity [3]. Larval dipteran evidence provides information on the length of time those insects have been present on a body postmortem (i.e., how long the resource has been colonized) which, excluding cases of myiasis, does not always correspond to the actual time since death [3,4]. For this reason, forensic entomologists often use the term minimum postmortem interval (PMI_min_) or the minimum amount of time elapsed since death as indicated by insect development [1]. Death scenes can be highly variable in their circumstances and insect colonization patterns immediately following death cannot always be assumed. For example, the arrival of insects can be delayed if a death occurs indoors [5], if the decedent is wrapped in cloth or like material [6], if the remains are altered by chemicals [7], or if the remains are burned [8]. Therefore, we use the term post-colonization interval (PCI) here when we refer to the time from insect colonization until discovery of the body [3].

The primary insect colonizers of decomposing bodies are blow flies (Diptera: Calliphoridae) [9,10]. The greatest influence on blow fly larval development is temperature, since insects are poikilothermic [1]. Therefore, the amount of time required for calliphorid larvae to develop depends on environmental temperature. In order to standardize development time for temperature dependent development, temperature data are commonly converted to accumulated degree hours (ADH) using thermal summation models [11,12,13]. However, there are many biotic and abiotic factors that can influence larval development, thus making it difficult to accurately estimate the initial time of colonization [14]. For example, rain [15], drugs [16,17,18], predation [19], larval sex [20], geographic location and population genetics [21,22], tissue type [23], and bacteria [24] can all significantly affect larval development by either increasing or decreasing the time required for carrion flies to complete their life cycle.

With all of this potential for variability at death scenes, the Daubert standard [25] and the 2009 National Academy of Sciences Report [26] suggest that laboratory and field variability studies are important to understanding how different factors affect insect development, such as the latter in this paper. Establishing a better understanding of the naturally occurring variability and factors that influence development is necessary to improve evidence collection and analysis techniques that can estimate any indexed concept, such as PCI or PMI, as accurately as possible [3,27]. Investigators at the scene may only collect the largest larval specimens from a body, on the assumption that they are the most developed, and, therefore, could be missing essential information for developing a more concise PMI estimate—even though protocols suggest collecting larvae of variable sizes [1,2,4,28]. Studies have shown that a larval mass can contain multiple blow fly species with abundances that change over time [29]. By only collecting the largest specimens, entire species that are potentially further along in their life cycle (i.e., older) but smaller in size could go unnoticed and uncollected, and thus an inaccurate estimate of the PCI would be generated based solely on the largest but perhaps not oldest larvae. For example, at 25 °C, a 10 mm *Cochliomyia macellaria* (Fabricius) (Diptera: Calliphoridae) larva would indicate 58 h [30] while a 9 mm *Phormia regina* (Meigen) (Diptera: Calliphoridae) larva would indicate 66 h [31]. Field studies like this one are important for investigating how different larval sizes (and within an instar) may lead to variable PCI estimates.

Another common practice in forensic entomology is to use the nearest, certified weather station to the death scene and previously published larval development data in order to determine insect developmental time [1,2]. While a weather station is the best source for historically accurate temperature data, it cannot be assumed that the station accurately reflects the microhabitat temperatures of a scene that is not near the said weather station [14,32]. Temperature variation between the death scene and weather station could potentially affect the accuracy of PCI estimates.

The objective of this study was to conduct a field study using multiple vertebrate carcasses to assess variation in PCI estimates derived from different temperature sources and published developmental data for two Calliphoridae species. The goal was not to build predictive models for PMI based on calliphorid life history traits, as has been done in other important studies [33,34,35], but rather, to explore how collecting multiple larval sizes of calliphorid species and using temperature data from multiple sources affected the range of PCIs that were calculated using different data sources. Our overall goal was to explore how multiple sources of variation in larval size and sources of temperature (as discussed by Amendt [4]) affected PCI calculations/estimates in a field study. ADH ranges were derived from temperature data sets acquired from instruments located at varying distances from the carcasses (i.e., individual carcass (0.90 m), microhabitat (0.90 m average of six carcasses), local (1.63 km), regional (7.61 km)), and used to calculate PCI estimates in order to evaluate how temperature sources influence ADH associated PCI estimates. We hypothesized that different Calliphoridae species would colonize the resource at different times resulting in larval size variation on and among carcasses, and that the temperature source nearest the carcasses (microhabitat) would provide temperatures most similar to what the larvae experience. Therefore, we predicted that different species, and larval sizes, would yield different PCI estimates and that the data from the closest temperature source to the carcasses would result in the most accurate PCI estimate. However, we also predicted that PCI estimates, regardless of temperature source utilized, would differ from the actual PCI due to differences in the published laboratory conditions (where developmental data were generated) and the field study conditions.

## 2. Materials and Methods

### 2.1. Study Site

The study was conducted from 19–27 August 2014 at Purdue University’s Forensic Entomology Research Compound in West Lafayette, Indiana (40°25′36.0″ N, 86°56′57.0″ W). Six (three male and three female) swine carcasses (*Sus scrofa* L.), averaging 19.65 kg (± 5.44 SD) and approximately 80.0 cm in length, were used as surrogates for human decomposition [1,36]. Carcasses were purchased from a local swine farm post-euthanization and therefore exempt from university Institutional Animal Care and Use Committee (IACUC) review. Carcasses were immediately placed inside two secured plastic bags during transport to the site (approximately 4 h), as previously described [37,38,39,40]. The carcasses were exposed along two East-West running transects approximately 50 m apart in a grassy field surrounded by deciduous forest. All carcasses were oriented on their left side with heads directed north and placed on top of stainless steel powder-coat painted grates (~1.0 m^2^, ~5.0 cm^2^ openings) located on the ground to allow for lifting the individual carcasses during sample collection. Cages (~1.0 m^3^) constructed of chicken mesh wire and treated lumber were secured over the carcasses and the grates to prevent vertebrate scavenging. Insect communities were sampled every 12 h at approximately 700 h and 1900 h for eight consecutive days (see *Larval Collections and Measurements* below), which was the amount of time required for the carcasses to fully decompose to only bones and skin, and for the dipteran larvae to enter the post-feeding dispersal stage.

### 2.2. Temperature

**Figure 1:**
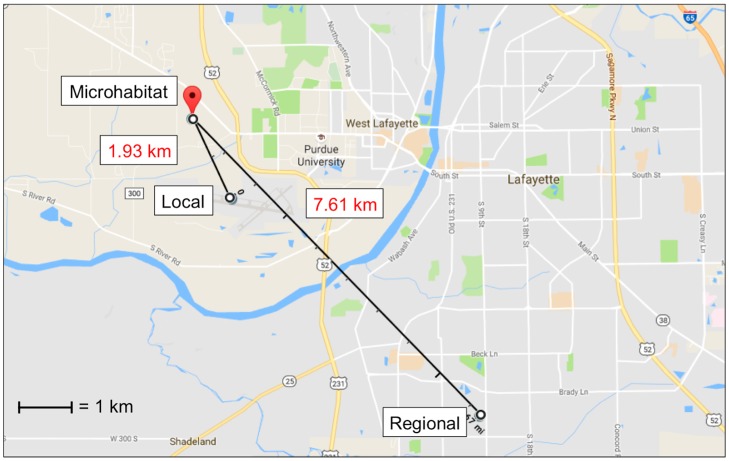
Map showing the location of and distances between the microhabitat (HOBO temperature loggers), local (Purdue University Airport weather station), and regional (Lafayette weather station) temperature sources. The microhabitat was the average of temperatures measured at each of the six carcasses. (Map: Google, Google).

Ambient temperature at each carcass was recorded every 15 min using one HOBO^TM^ temperature logger (Onset Computer Corporation, Bourne, MA, USA) per carcass attached to the east side of each anti-vertebrate scavenging cage approximately 0.90 m off the ground. The individual temperature source refers to the corresponding temperature logger for each carcass at 0.9 m distance. Temperature data from all six loggers were averaged to determine the temperature of the microhabitat source. Local source temperature data were acquired from the Purdue University Airport (40°24’48.5” N, 86°56’26.1” W) weather station, located 1.63 km southeast of the study site. Regional source temperature data were acquired from a Lafayette, Indiana weather station (MAU199) located 7.61 km southeast of the study site (40°22’40” N, 86°53’13” W) (Figure 1). Both local and regional sources recorded temperature at least once every hour throughout the study.

### 2.3. Larval Collections and Measurements

**Figure 2:**
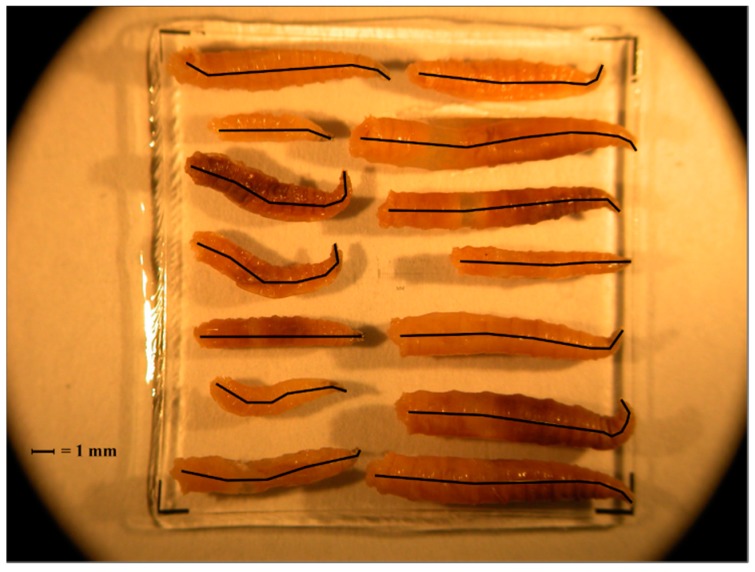
Example of third instars photographed on a micrometer slide and measured in ImageJ. Black lines indicate the midline that was measured in order to determine length of the larva.

At each sampling period, blow fly larvae were collected using a single cotton-tipped swab (Puritan Medical Products, Guilford, ME, USA) from three different areas of a single larval mass located on the carcass, and stored at −20 °C. Larval instars were determined based on the number of spiracular slits, as discussed elsewhere [41,42]. Third instars were identified to species level using published taxonomic keys for larvae [43,44] and then stored in 100% molecular grade ethanol. *Cochliomyia macellaria* and *P. regina* were used for PCI analyses due to specimen abundance and availability of previously published development data [30,31]. Each larva was photographed on a 2 mm micrometer slide and each body length was measured to the nearest 0.01 mm in ImageJ 1.46r [45]. This was done by setting the scale using the micrometer and measuring a line drawn through the larval midline while taking care to follow the curvature of each specimen (Figure 2).

### 2.4. ADH Ranges and Post-Colonization Intervals

All ADH ranges and PCIs were determined for third instars, since they were the most developed larvae collected and provide the most reliable identification using morphology for Calliphoridae larvae of forensic relevance [46]. Two ADH value sets (min, median, max) for *C. macellaria* and *P. regina* were calculated: “expected” ADH ranges using published laboratory-reared larval developmental data sets [30,31] and “actual” ADH ranges using temperatures recorded by the spatially distinct temperature sources (see *Temperature* section above for more details). Expected ADH ranges are those taken from the published data sets [30,31] that represent the expected ADH necessary for larvae of each species to reach the third instar stage or certain third instar lengths. Actual ADH ranges presumably reflect the temperature conditions of the larvae during decomposition for a given source. Actual ADH values for each species were then compared to expected ADH values. PCI estimates were also derived from the expected ADH ranges calculated for each temperature source and compared to the actual PCIs.

**Figure 3:**
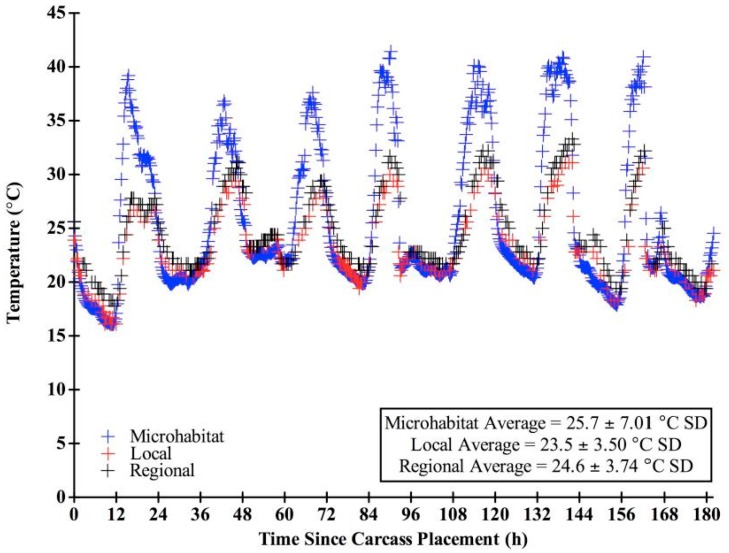
Temperature (°C) throughout decomposition for the microhabitat, local, and regional sources. Microhabitat data points are every 15 min while local and regional data points represent hourly temperatures.

We chose “expected” blow fly developmental data sets based on the range of developmental information provided in the literature and in concordance with our field conditions. Only studies that included: (1) developmental rates of the species; (2) associated larval length data, and (3) temperatures reflected in our field study were used in our analysis. Several other data sets are available, but were not utilized here due to a lack of larval length data [47,48,49]. Other studies utilized temperatures not consistent with those measured in this study [19,49,50,51,52,53]. For instance, data sets with hourly ranges for larvae reared at 25 °C were used for both species, since the overall average temperature recorded at the carcasses throughout decomposition in this study was 25.7 °C (± 7.01 SD) (Figure 3).

Actual ADH ranges were calculated by adding the hourly temperatures minus the minimum developmental threshold for each species, starting at time of carcass placement. The minimum developmental threshold was determined from previous literature: 10 °C for *C. macellaria* [19,50] and 6 °C for *P. regina* [54]. The minimum (min) and maximum (max) of actual ADH ranges match the time points when third instars were initially collected (min) and when they were last present for collection (max) for each carcass. In order to assess PCI variability, actual ADH ranges were calculated for each temperature source: the individual carcasses, the microhabitat, local, and regional temperature data. Temperature loggers of individual carcasses were used to calculate ADH ranges and PCIs in order to assess variability within the microhabitat and among replicates. Time point zero (0) represented the time when the carcasses were placed in the field, which corresponded to when the carcasses were initially exposed to potential insect colonization—approximately 4 h postmortem.

Third instar length was also utilized to calculate expected ADH values, as length can be an additional measurement used in forensic investigations. In order to understand how PCIs varied with larval length, we calculated the average 10th, 50th, and 90th percentile lengths of third instars collected from all carcasses for each calliphorid species. From these lengths, the associated ADH values were determined by visual assessment of previously published 25 °C growth curves [30,31] and minimum thresholds of 10 °C for *C. macellaria* [19,50] and 6 °C for *P. regina* [54]. Length data from previous developmental data sets were only available in line graph form [30,31], thus length to development time conversions were completed by extrapolating the length value to the growth curve above time using published recommendations of Wells and Lamotte [55] for this kind of data set. Extrapolation was to the nearest 0.25 mm.

PCI estimates were calculated as the hour ranges, for each temperature source, that correspond to the expected ADH values derived from third instar developmental stage and length data sets [30,31].

### 2.5. Statistical Analysis

Repeated measures analysis of variance (rm-ANOVA) was conducted to compare differences in temperature among the three spatially distinct weather data sets (microhabitat, local, regional) and among the individual carcasses [56]. Minimum, median, and maximum actual third instar ADH values among temperature sources were compared using an ANOVA. A paired t-test was performed to compare temperature differences between the local and regional sources over time, and between the actual and expected minimum, median, and maximum PCIs derived from the individual carcass temperatures. A Spearman Rank test was used to test for a correlation in the relative abundance of *C. macellaria* and *P. regina* third instars. Further, the coefficient of variation (CV) was used to represent temperature variability at each source and to represent the third instar lengths at each sampling time point for each species. All statistical tests were performed using SAS Studio 3.5 [57], and all *p*-values were considered significant with alpha ≤ 0.05.

## 3. Results

### 3.1. Temperature

There were significantly different (F = 10.22, df = 184, *p* < 0.0001) temperature means (± SD) among the microhabitat, local, and regional sources, which were 25.7 (± 7.01) °C, 23.5 (± 3.50) °C, and 24.6 (± 3.74) °C, respectively. Microhabitat temperatures were more variable (CV = 0.27) than both local (CV = 0.15) and regional (CV = 0.15) weather station temperatures. There was little variation between temperatures recorded at the local and regional sources; however, temperature differences between local and regional over time were significant (t = −12.19, df = 182, *p* < 0.0001). All three temperature sources recorded similar daily minimum temperatures (microhabitat: 19.5 ± 1.89 °C SD, local: 19.9 ± 2.06 °C SD, regional: 20.7 ± 1.40 °C SD). However, the microhabitat consistently recorded higher daily maximum temperatures (39.1 ± 1.65 °C SD) than the local (29.5 ± 1.56 °C SD) and regional (31.1 ± 1.89 °C SD) sources (Figure 3). There were significant mean temperature differences among the six carcasses (F = 38.31, df = 187, *p* < 0.0001). On average, temperature loggers of the western most carcass (N = 1: 25.4 ± 6.81 °C SD) and the eastern most carcasses (N = 2: 25.1 ± 6.53, 24.8 ± 5.64 °C SD) recorded lower temperatures than those in the center of the field (N = 3: 25.8 ± 7.32, 27.0 ± 9.38, 26.4 ± 8.38 °C SD).

### 3.2. Calliphoridae Larvae

**Figure 4:**
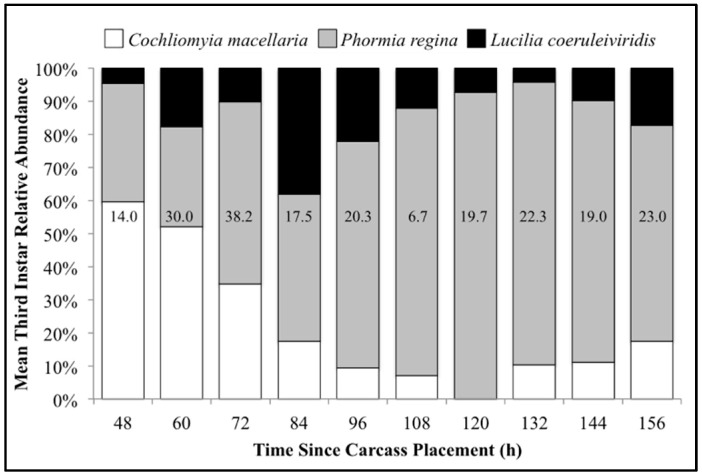
Mean relative abundance of all third instars collected throughout decomposition: *Cochliomyia macellaria*, *Phormia regina*, and *Lucilia coeruleiviridis*. Numbers on each column represent the average number of third instars (all species) collected at that time point.

**Figure 5:**
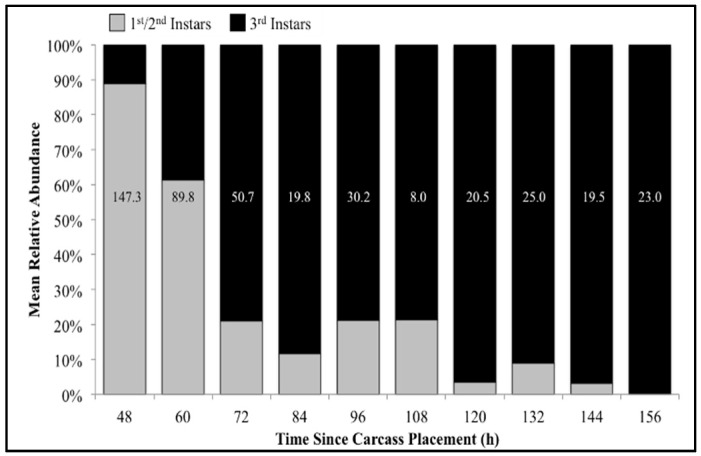
Mean relative abundance of first/second (pooled) and third instars of all species collected throughout decomposition. Numbers on each column represent the average number of larvae (all instars) collected at that time point.

The number of carcasses sampled at each time point varied with larval mass presence or absence and resource quantity remaining among carcasses. For example, larval masses were still present on the western most carcass at 156 h following placement while, at the same time, all the larvae from the other carcasses had already migrated away from the body to begin pupation. Calliphoridae egg clutches were first observed on two carcasses in the center of the field approximately 20–30 min after placement, while all other carcasses were colonized no more than 12 h later. Third instars were first collected as early as 48 h after carcass placement and as late as 156 h. A total of 904 third instars were identified: 258 *C. macellaria,* 509 *P. regina,* and 137 *Lucilia coeruleiviridis* (Macquart) (*Diptera: Calliphoridae*) (Table S1). The relative abundances and additional data of each species can be found in our recent publication [58]. All third instar *C. macellaria* and *P. regina* were measured and used for ADH and PCI calculations. *Lucilia coeruleiviridis* was not utilized due to low abundance and lack of developmental data in the literature. The greatest number of *C. macellaria* third instars was recorded within 48–60 h following carcass placement. The abundance of *P. regina* increased shortly thereafter (72 h) and then decreased (84–108 h) before increasing again (120 h) (Figure 4). *Cochliomyia macellaria* third instar average relative abundance was greatest at 48 h (59.51%), while *P. regina* relative abundance remained above 60% for most of decomposition from 84–156 h (Figure 4). There was a significant negative corrleation between *C. macellaria* and *P. regina* third instar average relative abundance over time (ρ = −0.9, *p* = 0.0002). The average relative abundance of third instars, compared to first and second instars, generally increased over time (Figure 5).

**Figure 6:**
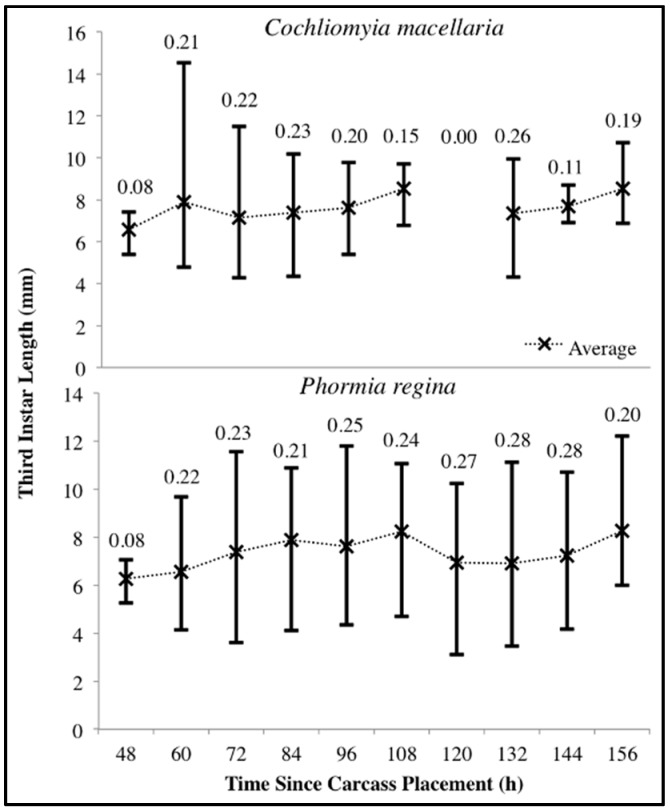
Range of *Cochliomyia macellaria* and *Phormia regina* third instar body length throughout decomposition. Upper and lower limits represent the maximum and minimum lengths, respectively. Average lengths are marked with an “X”. Coefficient of variation is noted above each time point. No *C. macellaria* third instars were collected at 120 h following carcass placement.

For both species there was substantial variability in third instar body lengths. The overall range of *C. macellaria* third instar lengths was 4.28–14.53 mm and 3.11–12.22 mm for *P. regina*. Even within a single sampling point, there was substantial length variability for each species. The greatest ranges within a single sampling point were 4.80–14.53 mm at 60 h and 3.62–11.55 mm at 72 h for *C. macellaria* and *P. regina,* respectively. The average (± SD) length for each species, however, changed little over time (*C. macellaria*: 7.63 ± 0.63 mm; *P. regina*: 7.33 ± 0.68 mm). The variability, represented as the CV for each time point, in third instar length fluctuated over time for *C. macellaria*, but the variability in *P. regina* remained fairly consistent throughout decomposition (Figure 6). There were significant differences among the 10th, 50th, and 90th percentile lengths for *C. macellaria* (F = 99.63, df = 2, *p* < 0.0001) and *P. regina* (F = 22.79, df = 2, *p* < 0.0001) (Table 1 and Table 2).

### 3.3. ADH Ranges

**Figure 7:**
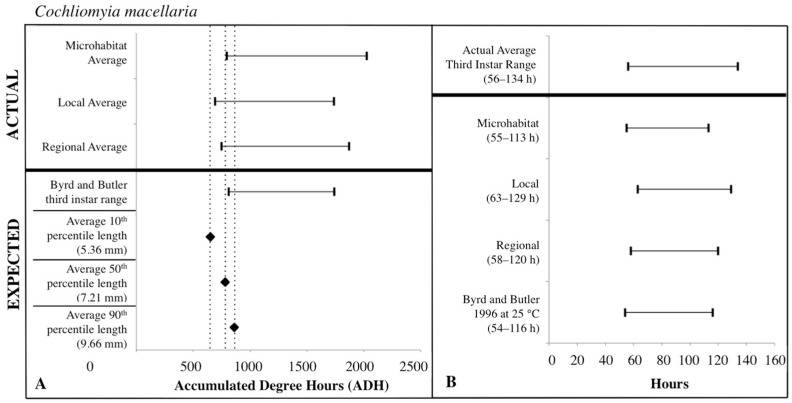
Comparison of expected accumulated degree hours (ADH) associated with *Cochliomyia macellaria* third instar developmental stage and length to actual third instar ADH ranges calculated for each temperature source: microhabitat, local, and regional. Actual ADH ranges represent the average range among carcasses. Black diamonds represent expected ADH values derived from 10th, 50th, and 90th percentile third instar lengths (**A**). Comparison of the average *Cochliomyia macellaria* third instar post-colonization interval (PCI as hours) to estimated PCIs for each temperature source (**B**). For both A and B: thin horizontal lines with vertical ends represent minimum-maximum ranges and values above the thick black line are actual while those below are expected or estimated based on different temperature sources or development data set.

**Figure 8:**
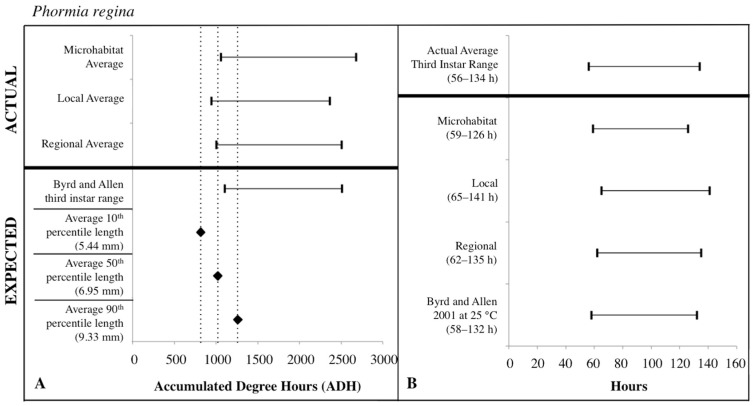
Comparison of expected accumulated degree hours (ADH) associated with *Phormia regina* third instar developmental stage and length to actual third instar ADH ranges calculated for each temperature source: microhabitat, local, and regional. Actual ADH ranges represent the average range among carcasses. Black diamonds represent expected ADH values derived from 10th, 50th, and 90th percentile third instar lengths (**A**). Comparison of the average *Phormia regina* third instar post-colonization interval (PCI as hours) to estimated PCIs for each temperature source (**B**). For both A and B: thin horizontal lines with vertical ends represent minimum-maximum ranges and values above the thick black line are actual while those below are expected or estimated based on different temperature sources or development data set.

The expected third instar ADH ranges from development data sets of Byrd and Butler [30] and Byrd and Allen [31] most often fell within the actual ADH range derived from the local temperatures for *C. macellaria* and regional temperatures for *P. regina*. For both species, microhabitat temperatures produced the widest ADH range (Figure 7 and Figure 8). The expected minimum ADH for both species, however, was greater than the actual minimum ADH for all temperature sources (*C. macellaria:* by 15, 120, and 63 ADH; *P. regina:* by 42, 157, and 99 ADH for microhabitat, local, and regional temperatures, respectively). The expected maximum ADH was less than the actual maximum ADH when microhabitat and regional temperatures were used with *C. macellaria* (by 284 and 130 ADH, respectively) and with microhabitat temperatures for *P. regina* (by 171 ADH) (Figure 7 and Figure 8). There were no significant differences for minimum, median, or maximum actual ADH among temperature sources for *C. macellaria* (F = 1.99, df = 3, *p* = 0.1473; F = 1.32, df = 3, *p* = 0.2947; F = 0.90, df = 3, *p* = 0.4604) or *P. regina* (F = 0.53, df = 3, *p* = 0.6649; F = 2.47, df = 3, *p* = 0.0914; F = 1.92, df = 3, *p* = 0.1592)*.*

The expected third instar ADH range encompassed more of the actual ADH range when larvae from *P. regina* were used. For *P. regina,* the expected third instar ADH range accounted for 86.8%, 88.9%, and 93.4% of the actual ADH range for microhabitat, local, and regional temperatures, respectively. For *C. macellaria,* the expected third instar ADH range was 75.7%, 88.5%, and 82.8% of the actual ADH range for microhabitat, local, and regional temperatures, respectively. Overall, the expected ADH ranges under-represented the actual ADH range, but did encompass the average actual ADH for all temperature sources (Table 1 and Table 2).

### 3.4. Post-Colonization Intervals

PCI estimates for each temperature source were calculated using the expected third instar ADH range for each species and temperature data for each source. There were no significant differences between the actual and estimated PCI minimum, median, and maximum hours derived from the individual carcass temperatures for either *C. macellaria* (t = −0.31, df = 5, *p* = 0.7681; t = 1.39, df = 5, *p* = 0.2224; t = 1.75, df = 5, *p* = 0.1406) or *P. regina* (t = −0.79, df = 5, *p* = 0.4640; t = 0.67, df = 5, *p* = 0.5310; t = 1.52, df = 5, *p* = 0.1901). Following the ADH calculations, the associated PCI estimates of *P. regina* expected ranges encompassed more of the actual PCI ranges. For *P. regina*, the third instar developmental stage based PCI estimates derived from the microhabitat, local, and regional temperature data accounted for 85.9%, 88.5%, and 92.3% of the actual PCI, respectively. For *C. macellaria,* the third instar developmental stage based PCI estimates derived from the microhabitat, local, and regional temperature accounted for 73.1%, 84.6%, and 79.5% of the actual PCI, respectively. Overall, microhabitat temperatures produced the narrowest PCI estimates and were the least accurate source for both species. Local temperatures produced the most accurate PCI estimate for *C. macellaria*, while regional temperatures were most accurate for *P. regina* (Figure 7 and Figure 8; Table 3 and Table 4)*.*

For third instar length-based estimates and previous developmental data sets [30,31], mean (± SD) larval lengths at the 10th percentile (*C. macellaria*: 5.36 ± 0.36 mm, *P. regina*: 5.44 ± 0.92 mm) for both species produced expected ADH values less than the actual minimum ADH for all temperatures sources. The 50th percentile lengths (*C. macellaria*: 7.21 ± 0.74 mm SD, *P. regina*: 6.95 ± 1.18 mm SD) of both species fell within local and regional temperature ADH ranges and just under the microhabitat minimum ADH. The 90th percentile length (*C. macellaria*: 9.66 ± 0.42 mm SD, *P. regina*: 9.33 ± 0.90 mm SD) for both species fell within the range from all sources. For both species, the expected ADH associated with the 50th percentile length was similar to the actual microhabitat and regional minimum ADH values (Figure 7 and Figure 8, Table 1 and Table 2).

Similar to the ADH ranges, the average length-based PCI estimates tended to underestimate the actual PCIs. For both species, 10th percentile lengths underestimated the actual PCI for all temperature sources (*C. macellaria:* by 5–13 h, *P. regina:* by 6–13 h). PCI estimates derived from 50th percentile lengths and local and regional temperatures fell within the actual PCI for both *C. macellaria* and *P. regina*, however, individual and microhabitat temperatures underestimated the actual PCI (*C. macellaria:* by 2–4 h, *P. regina:* by 1–2 h). All PCI estimates derived from 90th percentile lengths, regardless of species or temperature source, fell within the actual PCI (Table 5 and Table 6).

## 4. Discussion

Recently, the forensic science community has increased efforts to recognize the amount of variability in specific disciplines (e.g., trace evidence) and develop error rates [26]. Yet, it is still relatively unknown how the accuracy of using a calliphorid developmental data set to estimate a PCI varies depending on temperature source. Utilizing weather station data distant from the scene can be problematic, as possible temperature variations between the location of the station and the death scene could result in an inaccurate calculation of insect developmental time [14,32]. Archer [59] examined the accuracy of collecting scene temperatures after body removal and retrospectively correcting weather station data. In that study, often the minimum PMI estimates improved following correction, but temperature collection after body discovery is not always possible. Archer [59] urged caution when correcting data, since weather can change significantly over a short period of time. Improvement of PMI estimates was highly variable among correlation periods and in a few instances, temperature correction was associated with a decrease in estimated decomposition time accuracy. Additionally, in that excellent study only hypothetical insect data were analyzed [59].

Monthei [51] conducted a similar study to ours in Virginia in which accumulated degree days calculated from temperatures at the carcass (ambient, inside the cage, head, thorax, abdomen) and from two weather stations at different distances from the site (5.63 and 10.46 km) were used along with two *P. regina* developmental data sets [31,49] to estimate PMIs. The author reported that, when using Byrd and Allen’s data set [31], the furthest weather station from the study site produced the most accurate PMI estimate. When utilizing Anderson’s data set [49], however, temperatures from the head of the carcass produced the most accurate PMI estimate. Monthei postulated that this could be due to the fact that Anderson’s data came from larvae reared in masses while Byrd and Allen kept a ratio of 1.5 larvae per 1.0 g of pork [51]. Indeed, the role of larval mass heat from thermogenesis may both increase and stabilize temperatures that are best reflected in temperature conditions from weather stations; this would be a fruitful area of research comparing multiple locations in different geographic regions.

Contrary to our hypothesis, using temperature sources nearer to the carcasses did not result in increased expected ADH range accuracy or the associated estimates of the PCI. ADH ranges determined using published developmental data sets were surprisingly more similar to the actual third instar ADH ranges when weather station temperatures were used rather than temperatures from the scene. The expected ADH ranges under-represented the actual range when microhabitat temperatures were used: the ADH range using microhabitat temperatures was greater than the weather station ranges. This wider ADH range at the microhabitat may be explained by the temperature loggers being located closer to the ground (and thus exposed to increased radiant and reflective heat) than the weather stations, resulting in higher temperatures and thus more rapid accumulation of degree hours [1]. The greatest difference in temperatures between the sources occurred during the afternoon, when temperatures were highest. Additionally, developmental time is longer for *C. macellaria* and *P. regina* under cyclic temperatures than at a constant temperature [19]. This could explain why the expected ADH ranges under-represented the actual ADH ranges.

Regardless of the temperature source used to calculate ADH ranges for use in PCI estimates, we hypothesized that the artificial circumstances (i.e., controlled growth chamber) of the developmental data sets would result in differences in the estimated PCIs [30,31] compared to the actual PCIs based on 12 h interval field observations and sample collections [1]. Constant temperature conditions and other variables (e.g., humidity) of lab conditions are very different from field conditions where temperatures naturally fluctuate and development can be influenced by additional abiotic variables (e.g., precipitation). Our findings suggest that there were fewer extreme fluctuations in temperature from the weather stations than at the microhabitat, making it more similar to the laboratory setting where conditions were constant. These two sources of error—utilizing expected ADH ranges from data sets from conditions different than those experienced by larvae at the scene, and weather station temperatures that vary from those at the scene—may be canceling each other in PCI estimates: one may overestimate while the other underestimates development time [60].

Another notable finding was the variation in species and third instar length. This supports the importance of thorough evidence collection in order to utilize as much information available from a death scene as possible. The largest larvae may be the most developed and therefore an initial colonizer, but the presence of smaller third instars, of either the same or different species, could indicate multiple oviposition events that could help develop a more complete timeline of the post-colonization activity. Third instars were present for a wide range of time (hours), yet the relative abundances of this life stage compared to other instars shifted throughout decomposition. There was an increase in relative abundance of first and second instars at 96 h and then an increase in abundance of third instar *P. regina* at 120 h, suggesting a second oviposition event occurred later in the decomposition process [13]. Considering smaller third instars of another species collected from a body could be informative, as different species colonize at different times following death. Further, *P. regina* is known as a later colonizer during carrion decomposition, compared to *L. coeruleiviridis*, *C. macellaria*, and *Chrysomya rufifacies* (Macquart) (Diptera: Calliphoridae) [29], and prefers a resource that has been previously colonized [61]. We initially observed a high average relative abundance of *C. macellaria* third instars (59.51%) early on (48 h) followed by an increase in *P. regina* third instar average relative abundance (55.01% at 72 h) (Figure 4). Using the 50th and 90th percentile lengths of third instars was also effective at indicating the lower ADH and PCI range, but failed to encompass the upper ADH and PCI ranges. A possible explanation could be found in the preservation method. Specimens were frozen and then placed in ethanol, which could have led to shrinkages and underestimated ranges based on larval length [62]. It would be useful to replicate this study and compare PCI estimates derived from larval body length among different preservation methods. This knowledge would be valuable as experience and technique varies among investigators, and it is not uncommon for an entomologist to receive poorly preserved specimens.

Finally, the Calliphoridae collected during our study are similar to the species reported for a previous study conducted in the same geographic location [63]. This previous study also used swine carcasses and found *P. regina* and *L. coeruleiviridis* as colonizers (evident by larvae present on the carcasses) with additional blow fly species present as adults: *C. macellaria, Hydroatea leucostoma* (Fabricius) (Diptera: Calliphoridae), and *Pollenia rudis* (Fabricius) (Diptera: Calliphoridae) [63]. *Phormia regina* is considered a cold weather species, while *C. macellaria* is more abundant in warmer regions, however, both are common postmortem colonizers [30,31]. Thus, our findings are consistent with previous descriptions of the distribution and the ecology of the species that we collected in this study.

## 5. Conclusions

This study demonstrates that there is substantial variability in Calliphoridae species occurrence and larval development during carcass decomposition that can influence ADH-based estimates of the PCI. It further shows that the temperature source for forensic investigations using insect evidence can be highly variable, depending on the spatial location of the weather station or temperature sensor. The goal of this study was not to build predictive models for PCI or PMI based on calliphorid life history traits; however, these data do provide important insight into the potential abiotic and biotic variability that may influence the use and interpretation of calliphorid larvae evidence collected from entire carcasses (as human surrogates) in a natural habitat. Understanding these sources of variability in a natural environmental setting has the potential to inform both existing and future PMI predictive model building efforts [33,34,35]. Indeed, the importance of refining existing, or developing new, predictive models of PMI will move the discipline of forensic entomology forward and this study is only one effort to support these important endeavors. Additional studies are needed to more fully understand how variation in both biotic and abiotic factors among habitats and ecoregions can be used to develop broadly relevant and useful models in forensic entomology.

## Figures and Tables

**Table 1:** Actual and expected minimum (Min), median (Med), and maximum (Max) accumulated degree hours (ADH) associated with *Cochliomyia macellaria* development, using a minimum developmental threshold of 10° C, based on third instar range (time present on carcasses) and body lengths representing the 10th, 50th, and 90th percentiles. White and green areas represent actual values while gray areas represent expected values [30].

	**Third Instar Range (h)**															**10th Percentile**		**50th Percentile**		**90th Percentile**	
	Byrd and Butler 1996			Individual			Microhabitat			Local			Regional			Length (mm)		Length (mm)		Length (mm)	
Carcass	Min	Med	Max	Min	Med	Max	Min	Med	Max	Min	Med	Max	Min	Med	Max		ADH		ADH		ADH
1	810	1275	1740	727	1575	2423	718	1603	2487	610	1371	2132	663	1482	2300	5.70	690	7.40	780	10.08	870
2	810	1275	1740	706	1108	1510	718	1112	1506	610	946	1282	663	1025	1386	5.83	690	7.11	780	9.23	840
3	810	1275	1740	743	1247	1750	718	1180	1641	610	1016	1422	663	1098	1532	5.31	630	7.16	780	9.56	855
4	810	1275	1740	916	1523	2129	871	1471	2071	770	1279	1787	830	1376	1922	5.37	630	8.34	810	10.22	885
5	810	1275	1740	846	1560	2274	871	1621	2370	770	1387	2003	830	1494	2157	5.02	630	7.21	780	9.66	870
6	810	1275	1740	831	1411	1990	871	1471	2071	770	1279	1787	830	1376	1922	4.94	630	6.03	750	9.24	840
Mean	810	1275	1740	795	1404	2013	795	1410	2024	690	1213	1736	747	1309	1870	5.36	650	7.21	780	9.66	860
CV				0.10	0.14	0.17	0.11	0.15	0.19	0.13	0.15	0.19	0.12	0.15	0.19	0.07	0.05	0.10	0.02	0.04	0.02

**Table 2:** Actual and expected minimum (Min), median (Med), and maximum (Max) accumulated degree hours (ADH) associated with *Phormia regina* development, using a minimum developmental threshold of 6° C, based on third instar range (time present on carcass) and body lengths representing the 10th, 50th, and 90th percentiles. White and green areas represent actual values while gray areas represent expected values [31].

	**Third Instar Range (h)**															**10th Percentile**		**50th Percentile**		**90th Percentile**	
	Byrd and Allen 2001			Individual			Microhabitat			Local			Regional			Length (mm)		Length (mm)		Length (mm)	
Carcass	Min	Med	Max	Min	Med	Max	Min	Med	Max	Min	Med	Max	Min	Med	Max		ADH		ADH		ADH
1	1102	1805	2508	898	1973	3047	910	2011	3111	802	1779	2756	855	1890	2924	6.00	855	8.32	1216	10.34	1311
2	1102	1805	2508	919	1657	2395	910	1659	2408	802	1456	2109	855	1544	2233	5.28	836	6.60	874	9.61	1273
3	1102	1805	2508	935	1749	2563	910	1659	2408	802	1456	2109	855	1544	2233	4.94	836	6.77	1007	9.03	1254
4	1102	1805	2508	1156	1907	2657	1111	1855	2599	1010	1663	2315	1070	1760	2450	5.97	855	8.05	1197	9.97	1292
5	1102	1805	2508	1086	1968	2850	1111	2029	2946	1010	1795	2579	1070	1902	2733	6.50	874	6.90	988	9.28	1254
6	1102	1805	2508	1365	1942	2518	1406	2003	2599	1241	1778	2315	1311	1881	2450	3.93	646	5.04	836	7.76	1178
Mean	1102	1805	2508	1060	1866	2672	1060	1869	2679	945	1655	2364	1003	1754	2504	5.44	817	6.95	1020	9.33	1260
CV				0.17	0.07	0.09	0.19	0.09	0.11	0.19	0.10	0.11	0.18	0.10	0.11	0.17	0.10	0.17	0.16	0.10	0.04

**Table 3:** Actual and estimated *Cochliomyia macellaria* post-colonization intervals (PCIs) calculated using third instars and each temperature source, with a minimum developmental threshold of 10 °C. Min, med, and max represent the minimum, median, and maximum ADH value for each PCI. White areas represent actual values, while gray and blue areas represent estimated values [30].

	Third Instar Range (h)																	
	Actual			Byrd and Butler 1996			Individual			Microhabitat			Local			Regional		
**Carcass**	**Min**	**Med**	**Max**	**Min**	**Med**	**Max**	**Min**	**Med**	**Max**	**Min**	**Med**	**Max**	**Min**	**Med**	**Max**	**Min**	**Med**	**Max**
1	48	102	156	54	85	116	56	86	115	55	84	113	63	96	129	58	89	120
2	48	72	96	54	85	116	54	84	113	55	84	113	63	96	129	58	89	120
3	48	78	108	54	85	116	53	81	108	55	84	113	63	96	129	58	89	120
4	60	96	132	54	85	116	51	81	111	55	84	113	63	96	129	58	89	120
5	60	102	144	54	85	116	57	86	115	55	84	113	63	96	129	58	89	120
6	60	96	132	54	85	116	58	88	117	55	84	113	63	96	129	58	89	120
Average	56	91	134	54	85	116	55	84	113	55	84	113	63	96	129	58	89	120
Coefficient of Variation	0.12	0.14	0.17				0.05	0.03	0.03									

**Table 4:** Actual and estimated *Phormia regina* post-colonization intervals (PCIs) calculated using third instars and each temperature source, with a minimum developmental threshold of 6 °C. Min, med, and max represent the minimum, median, and maximum ADH value for each PCI. White areas represent actual values, while gray and blue areas represent estimated values [31].

	Third Instar Range (h)																	
	Actual			Byrd and Allen 2001			Individual			Microhabitat			Local			Regional		
**Carcass**	**Min**	**Med**	**Max**	**Min**	**Med**	**Max**	**Min**	**Med**	**Max**	**Min**	**Med**	**Max**	**Min**	**Med**	**Max**	**Min**	**Med**	**Max**
1	48	102	156	58	95	132	60	95	130	59	93	126	65	103	141	62	99	135
2	48	84	120	58	95	132	59	93	127	59	93	126	65	103	141	62	99	135
3	48	84	120	58	95	132	57	88	118	59	93	126	65	103	141	62	99	135
4	60	96	132	58	95	132	56	90	123	59	93	126	65	103	141	62	99	135
5	60	102	144	58	95	132	61	96	131	59	93	126	65	103	141	62	99	135
6	72	102	132	58	95	132	61	97	132	59	93	126	65	103	141	62	99	135
Average	56	95	134	58	95	132	59	93	127	59	93	126	65	103	141	62	99	135
Coefficient of Variation	0.18	0.09	0.11				0.04	0.04	0.04									

**Table 5:** Actual and estimated *Cochliomyia macellaria* post-colonization intervals (PCIs) calculated using 10th, 50th, and 90th percentile third instar lengths and each temperature source, with a minimum developmental threshold of 10 °C. Min, med, and max represent the minimum, median, and maximum hourly value for each PCI. All time values represent hours. White areas represent actual values, while gray and blue areas represent estimated values from published development data and body lengths [30].

	Actual	10th Percentile						50th Percentile						90th Percentile					
Carcass	Third Instar Range	Length (mm)	Byrd	Individual	Microhabitat	Local	Regional	Length (mm)	Byrd	Individual	Microhabitat	Local	Regional	Length (mm)	Byrd	Individual	Microhabitat	Local	Regional
1	48–156	5.70	46	46–47	46–47	54	49–50	7.40	52	53–54	52–53	60–61	56–57	10.08	58	61	59–60	67–68	63–64
2	48–96	5.83	46	45–46	46–47	54	49–50	7.11	52	53–54	52–53	60–61	56–57	9.23	56	57	57–58	65–66	60–61
3	48–108	5.31	42	42–43	43–44	49–50	46–47	7.16	52	53–54	52–53	60–61	56–57	9.56	57	56–57	58–59	66–67	62–63
4	60–132	5.37	42	41–42	43–44	49–50	46–47	8.34	54	56–57	55–56	63–64	58–59	10.22	59	57–58	61–62	68–69	64–65
5	60–144	5.02	42	44–45	43–44	49–50	46–47	7.21	52	53–54	52–53	60–61	56–57	9.66	58	61–62	59–60	67–68	63–64
6	60–132	4.94	42	45–46	43–44	49–50	46–47	6.03	50	51–52	50–51	58–59	54–55	9.24	56	60–61	57–58	65–66	60–61
Average	56–134	5.36	43	44–45	44–45	51	47–48	7.21	52	53–54	52–53	60–61	56–57	9.66	57	59	59	66–67	62–63
Coefficient of Variation		0.07	0.05					0.10	0.02					0.04	0.02				

**Table 6:** Actual and estimated *Phormia regina* post-colonization intervals (PCIs) calculated using 10th, 50th, and 90th percentile third instar lengths and each temperature source, with a minimum developmental threshold of 6 °C. Min, med, and max represent the minimum, median, and maximum hourly value for each PCI. All time values represent hours. White areas represent actual values, while gray and blue areas represent estimated values from published development data and body lengths [31].

	Actual	10th Percentile						50th Percentile						90th Percentile					
Carcass	Third Instar Range	Length (mm)	Byrd	Individual	Microhabitat	Local	Regional	Length (mm)	Byrd	Individual	Microhabitat	Local	Regional	Length (mm)	Byrd	Individual	Microhabitat	Local	Regional
1	48–156	6.00	45	45–46	45–46	51–52	48	8.32	64	65–66	65–66	70–71	67–68	10.34	69	69–70	68–69	76–77	72
2	48–120	5.28	44	44–45	44–45	49–50	47–48	6.60	46	45–46	46–47	52–53	48–49	9.61	67	66–67	67–68	73–74	70–71
3	48–120	4.94	44	43–44	44–45	49–50	47–48	6.77	53	52–53	53–54	59–60	56–57	9.03	66	65–66	66–67	72–73	69–70
4	60–132	5.97	45	43–44	45–46	51–52	48	8.05	63	62–63	64–65	69–70	67	9.97	68	66	67–68	74–75	71–72
5	60–144	6.50	46	47–48	46–47	52–53	48–49	6.90	52	54–55	52–53	58–59	55–56	9.28	66	67–68	66–67	72–73	69–70
6	72–132	3.93	34	39–40	37–38	41–42	38–39	5.04	44	46–47	44–45	49–50	47–48	7.76	62	65–66	63–64	69–70	66–67
Average	56–134	5.44	43	44	44	49–50	46–47	6.95	54	54–55	54–55	60	57–58	9.33	66	66–67	66–67	73–74	69–70
Coefficient of Variation		0.17	0.10					0.17	0.16					0.10	0.04				

## Supplementary Materials

The following are available online at http://www.mdpi.com/2075-4450/8/2/40/s1, Table S1: Number of larvae, and third instars specifically, collected per carcass at each sampling period.
