# Supplementary material for: Post-Colonization Interval Estimates Using Multi-Species Calliphoridae Larval Masses and Spatially Distinct Temperature Data Sets: A Case Study
Source: Insects. 2017 Apr 4;8(2):40. doi: 10.3390/insects8020040 (PMC5492054; doi:10.3390/insects8020040)
Supplement: Supplementary file 1 [file insects-08-00040-s001.pdf]

# Supplementary Materials: Post-Colonization Interval Estimates Using Multi-Species Calliphoridae Larval Masses and Spatially Distinct Temperature Data Sets: A Case Study

**Courtney R. Weatherbee, Jennifer L. Pechal, Trevor Stamper and M. Eric Benbow**

**Table S1.** Number of larvae, and third instars specifically, collected per carcass at each sampling period. Total larvae refers to all instars collected: first, second, and third. Larval data begins at 48 h since carcass placement, as that was the first sampling period when larval masses were present. "X"s indicate times when no larval masses were present for that carcass.

| Time Since Carcass Placement (h) | Carcass | Total Larvae | Total 3rd Instars Collected | *Cochliomyia Macellaria* 3rd Instars | *Lucilia coeruleiviridis* 3rd Instars | *Phormia Regina* 3rd Instars |
|---|---|---|---|---|---|---|
| 48 | 1 | 197 | 11 | 7 | 0 | 4 |
| | 2 | 106 | 24 | 7 | 0 | 17 |
| | 3 | 139 | 7 | 6 | 1 | 0 |
| | 4 | X | X | X | X | X |
| | 5 | X | X | X | X | X |
| | 6 | X | X | X | X | X |
| 60 | 1 | 98 | 56 | 35 | 1 | 20 |
| | 2 | 80 | 31 | 16 | 7 | 8 |
| | 3 | 199 | 44 | 24 | 5 | 15 |
| | 4 | 32 | 19 | 7 | 2 | 10 |
| | 5 | 39 | 15 | 5 | 5 | 5 |
| | 6 | 91 | 15 | 11 | 4 | 0 |
| 72 | 1 | 97 | 68 | 49 | 5 | 14 |
| | 2 | 43 | 25 | 14 | 5 | 6 |
| | 3 | 53 | 48 | 8 | 3 | 37 |
| | 4 | 22 | 21 | 5 | 0 | 16 |
| | 5 | 42 | 36 | 5 | 2 | 29 |
| | 6 | 36 | 31 | 8 | 7 | 16 |
| 84 | 1 | 21 | 19 | 6 | 0 | 13 |
| | 2 | 20 | 17 | 4 | 9 | 4 |
| | 3 | 23 | 19 | 4 | 11 | 4 |
| | 4 | 20 | 19 | 3 | 11 | 5 |
| | 5 | 18 | 16 | 1 | 0 | 15 |
| | 6 | 18 | 15 | 1 | 9 | 5 |
| 96 | 1 | 32 | 28 | 3 | 5 | 20 |
| | 2 | 35 | 33 | 2 | 3 | 28 |
| | 3 | 18 | 15 | 1 | 2 | 12 |
| | 4 | 17 | 15 | 1 | 10 | 4 |
| | 5 | 16 | 16 | 2 | 0 | 14 |
| | 6 | 61 | 15 | 2 | 4 | 9 |
| 108 | 1 | 10 | 8 | 0 | 0 | 8 |
| | 2 | 1 | 1 | 0 | 0 | 1 |
| | 3 | 7 | 6 | 1 | 1 | 4 |
| | 4 | 12 | 12 | 3 | 2 | 7 |
| | 5 | 11 | 8 | 0 | 0 | 8 |
| | 6 | 5 | 5 | 0 | 2 | 3 |
| 120 | 1 | 19 | 19 | 0 | 0 | 19 |
| | 2 | 11 | 11 | 0 | 0 | 11 |
| | 3 | 18 | 18 | 0 | 6 | 12 |
| | 4 | 14 | 14 | 0 | 0 | 14 |
| | 5 | 39 | 38 | 0 | 2 | 36 |
| | 6 | 22 | 18 | 0 | 1 | 17 |
| 132 | 1 | 19 | 19 | 2 | 0 | 17 |
| | 2 | X | X | X | X | X |
| | 3 | X | X | X | X | X |

| | | | | | | |
|---|---|---|---|---|---|---|
| | 4 | 22 | 22 | 4 | 1 | 17 |
| | 5 | 27 | 25 | 1 | 0 | 24 |
| | 6 | 32 | 23 | 2 | 3 | 18 |
| 144 | 1 | 23 | 23 | 2 | 3 | 18 |
| | 2 | X | X | X | X | X |
| | 3 | X | X | X | X | X |
| | 4 | X | X | X | X | X |
| | 5 | 16 | 15 | 2 | 1 | 12 |
| | 6 | X | X | X | X | X |
| 156 | 1 | 23 | 23 | 4 | 4 | 15 |
| | 2 | X | X | X | X | X |
| | 3 | X | X | X | X | X |
| | 4 | X | X | X | X | X |
| | 5 | X | X | X | X | X |
| | 6 | X | X | X | X | X |

© 2017 by the authors. Submitted for possible open access publication under the terms and conditions of the Creative Commons Attribution (CC BY) license (http://creativecommons.org/licenses/by/4.0/).
